# Social factors influencing behavioral intentions to vaccinate: personality traits and cues to action

**DOI:** 10.3389/fpsyg.2025.1481147

**Published:** 2025-01-23

**Authors:** Zeming Li, Xinying Sun

**Affiliations:** ^1^Department of Social Medicine, School of Public Health, Guangxi Medical University, Guagnxi, Nanning, China; ^2^Department of Social Medicine and Health Education, School of Public Health, Peking University, Beijing, China

**Keywords:** personality traits, cues to action, Five-Factor Model, health belief model, vaccination

## Abstract

**Objectives:**

This study integrates the Five-Factor Model (FFM) of personality traits with the Health Belief Model (HBM) to examine associations among personality traits, cues to action, and vaccination intentions.

**Method:**

An online survey was conducted in April 2021, with 2,098 participants (mean age = 31.22 years, SD = 8.29) completing the study. The questionnaire assessed HBM constructs and the FFM personality traits. Spearman correlation coefficients were used to evaluate associations among ordinal variables, while Structural Equation Modeling (SEM) explored complex relationships between latent variables.

**Results:**

The findings indicate that self-efficacy (β = 0.198) and perceived barriers (β = 0.515) exert the most significant direct positive influences on vaccination intentions. Cues to action, particularly recommendations from family members (β = 0.113) and doctors (β = 0.092), also significantly affect vaccination intentions. Notably, personality traits indirectly influence vaccination intentions through self-efficacy and perceived barriers. Furthermore, agreeableness most significantly affects family suggestions, while neuroticism strongly influences recommendations from authority figures and healthcare providers, with extraversion notably impacting suggestions from peers.

**Conclusions:**

The study highlights the influence of personality traits on cues to action, with neuroticism linked to authority influence, extraversion to peer influence, and agreeableness to familial influence. These findings emphasize the importance of incorporating individual differences into public health policies and vaccination promotion strategies. Future research should further explore the effects of diverse personality traits and community-specific profiles on vaccination behaviors to enhance intervention effectiveness.

## 1 Background

The global spread of coronavirus disease 2019 (COVID-19) has significantly impacted human health and economic development. In May 2023, the World Health Organization (WHO) declared that COVID-19 no longer constitutes a public health emergency of international concern (World Health Organization, 2023). However, vaccination remains a critical measure for preventing and controlling infectious diseases (Forni et al., 2021). Despite efforts by governments and the public to increase vaccination rates, initial acceptance of the COVID-19 vaccine has been hindered by safety concerns and misinformation, leading to widespread vaccine hesitancy (Fisher et al., 2020; Lazarus et al., 2021), which is regarded as one of the top 10 global health threats (Harrison and Wu, 2020). As viruses continue to mutate, regular vaccinations may be necessary to maintain herd immunity. Therefore, a deeper understanding of public attitudes and behaviors toward vaccines is essential for effectively addressing potential future outbreaks and providing essential insights into existing public health strategies. It can inform the development of more effective communication strategies and interventions, ultimately enhancing vaccination rates and safeguarding community health.

The Health Belief Model (HBM) explains individuals' motivation to adopt health-related behaviors (Rosenstock et al., 1988). HBM consists of four key components: Perceived threat of disease: this includes perceived severity and perceived susceptibility. Perceived severity refers to an individual's perception of the consequences of a disease, encompassing its adverse effects on physical health and psychological and social aspects, such as physical strength, appearance, work, and social life. Perceived susceptibility denotes an individual's assessment of the likelihood of contracting the disease. For instance, perceptions of the severity of COVID-19 and personal risk of infection significantly influence attitudes toward vaccination. Perceived benefits and barriers: this involves an individual's subjective evaluation of the advantages and disadvantages of adopting or refraining from a behavior. Perceived benefits relate to the protective effects of vaccination, which can enhance the public's intention to vaccinate. Conversely, perceived barriers, such as concerns about vaccine side effects, may contribute to vaccine hesitancy. Self-efficacy: similar to self-confidence, self-efficacy reflects an individual's belief in their ability to successfully adopt health behaviors by managing internal and external factors. This confidence directly impacts vaccination behavior. Cues to action: these are factors that prompt health behaviors, acting as the “last driving force” for behavioral change. According to Rosenstock's model, cues to action serve as triggers that motivate individuals to take action. Examples include health education through mass media, advice from healthcare professionals, and encouragement from family and social groups. The more numerous and authoritative these cues, the higher the likelihood of individuals adopting healthy behaviors.

**Figure 1:**
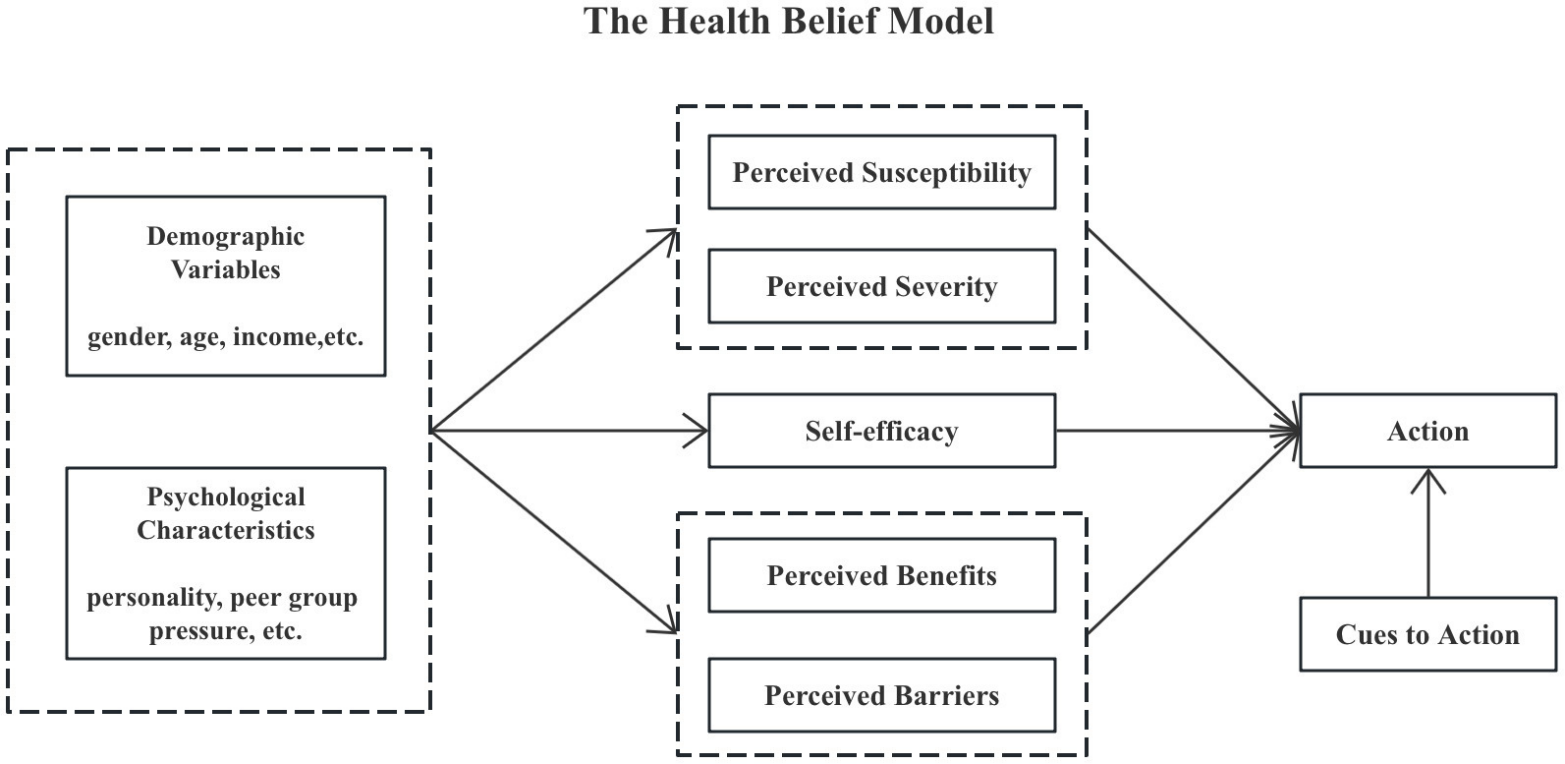
The Health Belief Model structure diagram.

The Health Belief Model (HBM) posits that health beliefs are cognitive and behavioral factors that influence health-related behaviors and catalyze behavioral changes. Figure 1 illustrates the structure of the HBM. Numerous studies have examined the factors affecting vaccination behavior and intention through the lens of health belief models to inform health education policies (Wong et al., 2021; Chen et al., 2021). However, significant research gaps remain. Most studies do not adequately address individual differences and lack empirical foundations for personalized health education strategies. This study aims to explore the factors influencing public intentions toward COVID-19 vaccination based on HBM. By identifying the specific effects of various personality traits on vaccine intentions, we aim to provide tailored recommendations for public health policy, enhancing efficiency and fostering the development of personalized health education strategies to promote vaccination effectively.

In the team's previous studies, correlations were found between personality traits and self-management behaviors, including medication, exercise and diet, in patients with type 2 diabetes (Li et al., 2020; Gao et al., 2021). Based on these findings, it can be inferred that the effectiveness and acceptance of chronic disease interventions may vary according to patients' personality traits.

Personality traits are relatively stable and unique patterns of psychological behavior based on innate biological and genetic qualities through interaction with the acquired social environment. Personality traits have important implications in determining an individual's thoughts, emotions, actions, and perceptions of health outcomes (Dubayova et al., 2009; Yamaoka et al., 1998; Chapman et al., 2007). To better understand the relationship between personality traits and health outcomes, research has explored how these five traits affect a range of physical and mental health conditions. The Five-Factor Model (FFM) of personality traits, proposed by McCrae and Costa in 1985, comprised five personality traits: Openness, Conscientiousness, Extraversion, Agreeableness, and Neuroticism (OCEAN) (McCrae and Costa, 1985). To summarize, neuroticism evaluates emotional stability, conscientiousness assesses an individual's attitude and self-control in goal-directed behavior, agreeableness measures attitudes toward others and quality of interpersonal orientation, openness measures willingness to accept novel concepts, and extroversion primarily measures the extent of interpersonal interaction among individuals (McCrae and John, 1992; Costa, 1995).

Although self-management behaviors for type 2 diabetes and vaccination behaviors may seem distinct, both involve individual health management and decision-making processes. Personality traits significantly influence how individuals receive health information, respond to interventions, and form attitudes toward vaccination. For instance, individuals with high levels of conscientiousness are more likely to adhere to health recommendations, including vaccination. Thus, understanding how personality traits affect chronic disease management outcomes can provide valuable insights for predicting and enhancing vaccination intentions.

Furthermore, personality traits play a crucial role in the construction of the Health Belief Model (HBM), especially in the context of vaccination. For example, individuals exhibiting high levels of neuroticism tend to demonstrate increased sensitivity to health risks, which may enhance their perception of susceptibility to vaccine-preventable diseases. In contrast, those with high conscientiousness typically exhibit greater self-efficacy, believing in their ability to understand the necessity of vaccination and take appropriate actions. This confidence increases their likelihood of adhering to health recommendations, including vaccination. Conscientious individuals often display higher self-control in their daily lives, making them more proactive in engaging in health management behaviors. Therefore, personality traits not only influence how individuals receive health information but may also modulate vaccination intentions by affecting perceptions of susceptibility and self-efficacy within the HBM framework.

In the context of interpersonal health education for vaccination, recommendations from various sources—such as healthcare providers, family members, and friends—are crucial (Gargano et al., 2015; Ellingson et al., 2023; Redmond et al., 2010). Engaging these stakeholders—doctors, family, friends, and supervisors—to provide encouragement and advice can be time-consuming and resource-intensive. Given that different personality traits exhibit distinct preferences, we hypothesize that individuals with varying personality characteristics may have specific preferences for advice from different roles. Based on this premise, we integrate the Five-Factor Model (FFM) with the Health Belief Model (HBM) to explore the feasibility of personalized health education in vaccination initiatives.

This study investigates how personality traits influence public intentions toward vaccination, grounded in the Health Belief Model. We categorize personality traits into five types based on the Five-Factor Model and explore the relationship between these traits and cues to action, as well as their effects on behavioral intentions through perceptual factors such as self-efficacy, susceptibility, perceived benefits, and barriers. Finally, we will summarize targeted vaccination intervention strategies based on personality traits, providing a scientific foundation for the development of future health education and intervention programs.

## 2 Materials and methods

### 2.1 Participants and procedures

The survey was conducted via the online platform WENJUANXING (wjx. cn) in April 2021. We employed a stratified random sampling method to select participants aged 18 and older, ensuring stratification by gender, age, and region. This methodology was chosen to accurately reflect these critical demographic variables, thereby enhancing the external validity of the study's findings. A total of 2,098 valid questionnaires were collected.

### 2.2 Measures

The questionnaire is structured into three sections: demographic information, which includes gender, age, education level, marital status, and per capita monthly household income, as well as health-related behaviors, specifically mask-wearing and hand hygiene. The inclusion of hand hygiene and mask-wearing variables is justified by their relevance to overall health behaviors during the pandemic. For hand hygiene, participants responded to three items, such as, “How often did you wash your hands before eating in the past month?” For mask-wearing, they answered two items, including, “How often did you wear a mask when going outside in the past month?” The frequency of these behaviors was categorized into five levels: seldom, sometimes, often, always, and every day.

The second part is a scale designed based on the HBM, using a Likert 5-point scale. Exploratory Factor Analysis (EFA) revealed a cumulative contribution of 56.42% for seven factors: (i) perceived severity contained four items (α = 0.790), (ii) perceived susceptibility contained four items (α = 0.714), (iii) perceived benefits contained three items (α = 0.639), (iv) behavioral barriers contained four items (α = 0.734), (v) self-efficacy contained four items (α = 0.809), (vi) cues to action contained four items (α = 0.715), and (vii) behavioral intentions contained three items (α = 0.761). The internal consistency of Cronbach's alpha coefficient for each subscale was above 0.6, with reliability in acceptable limits. The content of the questionnaire is shown in Appendix A.

The third part is the Chinese version of the Ten-Item Personality Inventory in China (TIPI-C), which was translated from English to Chinese by Chinese scholar Li JD based on the Ten-Item Personality Inventory (TIPI) (Li, 2013). Developed by Gosling et al., it was used in a college student population and tested for reliability, which can be used as a reliable and efficient tool for measuring the FFM of personality traits (Gosling et al., 2003). The TIPI-C consists of five subscales: Openness (O), Conscientiousness (C), Extraversion (E), Agreeableness (A), and Neuroticism (N). The internal Cronbach's alpha coefficients for the five subscales were 0.771 (N), 0.381 (C), 0.603 (A), 0.638 (O), and 0.592 (E).

Although some subscales of the questionnaire we utilized, such as perceived benefits and conscientiousness, displayed relatively low Cronbach's alpha values (0.639 and 0.381, respectively), these values may still be considered acceptable in certain contexts. Based on Nunnally and Bernstein's findings, they suggested that alpha values ranging from 0.60 to 0.70 can be deemed acceptable in exploratory research (Ponterotto and Ruckdeschel, 2007). Furthermore, low alpha values may reflect the diversity or complexity of the constructs rather than indicating the ineffectiveness of the scales. Additionally, while the TIPI-C demonstrates commendable brevity in evaluating personality traits, the low internal consistency (Cronbach's alpha) of some subscales is a recognized issue closely related to the scale's conciseness. According to relevant literature (Gosling et al., 2003), shorter scales may inadequately capture the complexity of certain personality traits, potentially leading to reduced internal consistency. Therefore, we acknowledge the low internal consistency of some subscales as a limitation of this study. Future research will aim to further validate and refine these scales, considering the use of more standardized personality trait measures to enhance the reliability of the assessments.

### 2.3 Quality control

Participants only completed the questionnaire once per ID number using a computer or smartphone through the link posted on WENJUANXING (wjx.cn). Questionnaires exhibiting uniform responses across all items or containing illogical answers were deemed invalid. A total of 2,098 valid questionnaires were returned after cleaning and collating the questionnaires.

### 2.4 Informed consent

This study protocol was reviewed and approved by the Biomedical Ethics Committee of Peking University (IRB00001052-20081). The questionnaire was conducted after respondents understood the survey and signed the informed consent.

### 2.5 Data analysis

Statistical analysis was performed through SPSS 26.0, and frequency percentage was used to describe participant characteristics. Spearman correlation analysis was used to check the correlation between variables. A correlation heat map was generated using R Studio to visually represent the relationships between variables, which is a widely used visualization tool that transforms regularized matrix data into colors, where each small square represents the correlation coefficient between variables. The structural equation model was constructed through Mplus 8.0, which is a statistical method to analyze the correlation between variables based on covariance matrices. In this study, SEM was constructed using the estimation of least squares.

The model fit was evaluated using several indices: the Comparative Fit Index (CFI), which serves as a statistical measure for assessing model fit; the Tucker-Lewis Index (TLI), another indicator of model fit that is similar to the CFI, with values closer to 1 indicating better fit. A model is deemed acceptable when both CFI and TLI exceed 0.90. Additionally, the Standardized Root Mean Square Residual (SRMR) measures the discrepancy between predicted and observed values, with values below 0.08 indicating a better fit (Hu and Bentler, 1999). The Root Mean Square Error of Approximation (RMSEA) is utilized to assess model fit, where values < 0.05 suggest good fit, values below 0.01 indicate a perfect fit, and values under 0.10 are regarded as acceptable (Steiger, 1990). The significance level for the analyses was set at *P* < 0.05.

## 3 Results

This study comprised 2,098 participants with a mean age of 31.22 ± 8.29 years, consisting of 53.10% males and 46.90% females. Most of the participants got married (61.92%) and held a bachelor's degree (82.84%). Their monthly family income per capita was medium-low, varying from 5,000 CNY to 9,999 CNY (32.32%), as shown in Table 1.

**Table 1:** Characteristics of the participants (*N* = 2,098).

**Characteristics**		***N***	**%**
Gender	Male	1,114	53.10
	Female	984	46.90
Age groups	18–29	928	44.23
	30–39	862	41.09
	40–49	238	11.34
	≥50	70	3.34
Education levels	High School and below	180	8.58
	Undergraduate	1,738	82.84
	Postgraduate and above	180	8.58
Marital status	Unmarried	799	38.08
	Married	1,299	61.92
Per capita monthly household (CNY)	< 5,000	527	24.98
	5,000–9,999	678	32.32
	10,000–14,999	407	19.40
	≥15,000	489	23.30
Past behavior	Vaccinated	860	40.99
	Unvaccinated	1,238	59.01

**Figure 2:**
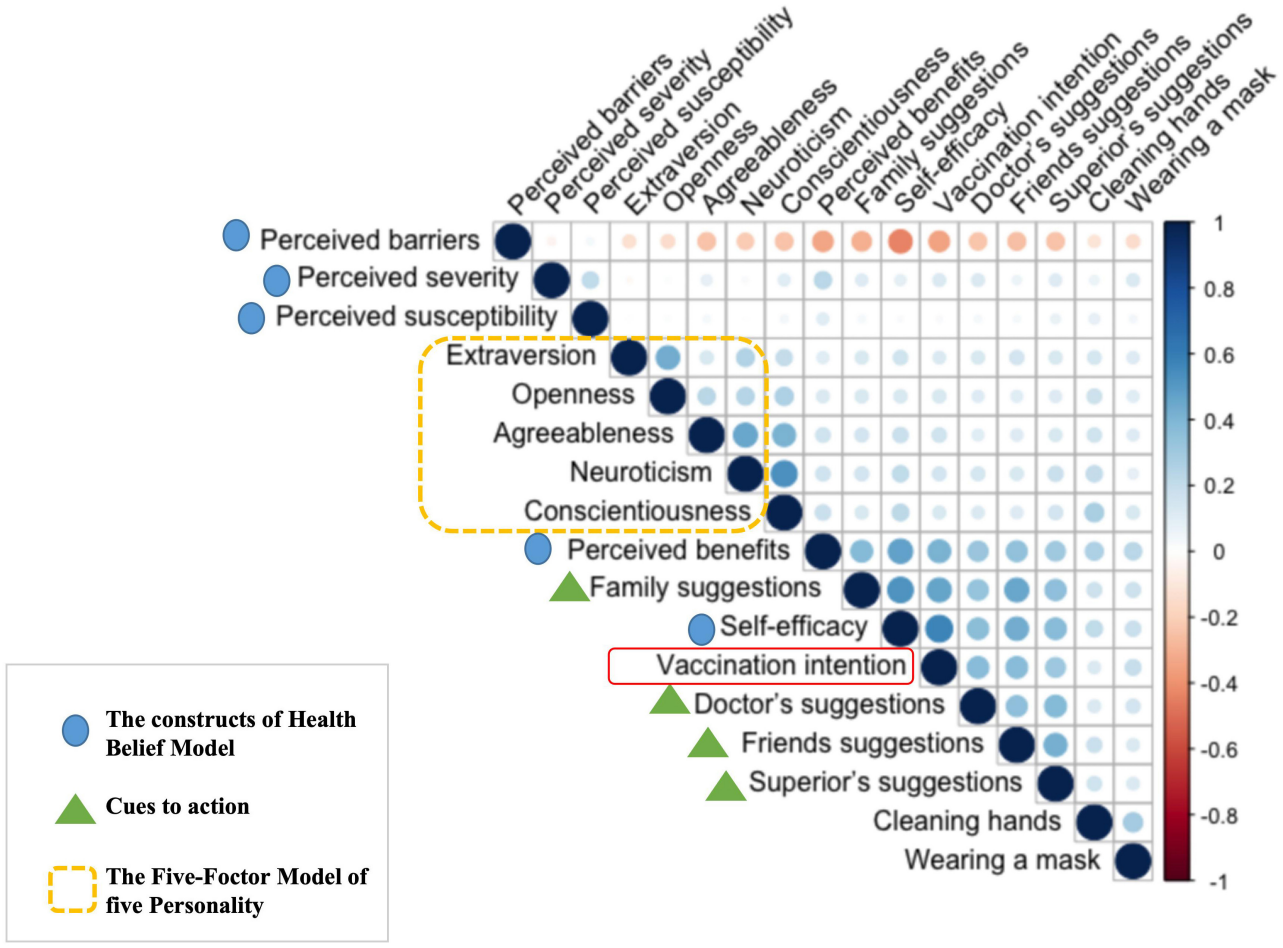
The correlation heat map of the variables of HBM and FFM.

This study, grounded in the Health Belief Model and the Five-Factor Model, examines the influence of various personality traits on the intention to receive the COVID-19 vaccination. Figure 2 illustrates the correlations among the variables. Pearson's correlation coefficient was employed to quantify these relationships, as depicted in the heat map. The color intensity reflects the magnitude of the correlation: darker shades indicate stronger correlations, with red representing negative correlations and blue denoting positive correlations. The survey results reveal that self-efficacy is the most significant positive factor influencing vaccination intention, as indicated by the darkest blue in the heat map. Following self-efficacy, cues to action—such as recommendations from family members, doctors, friends, and supervisors—demonstrate a strong positive correlation with vaccination intention. Furthermore, a positive relationship is observed between the five personality traits and the four cues to action.

**Figure 3:**
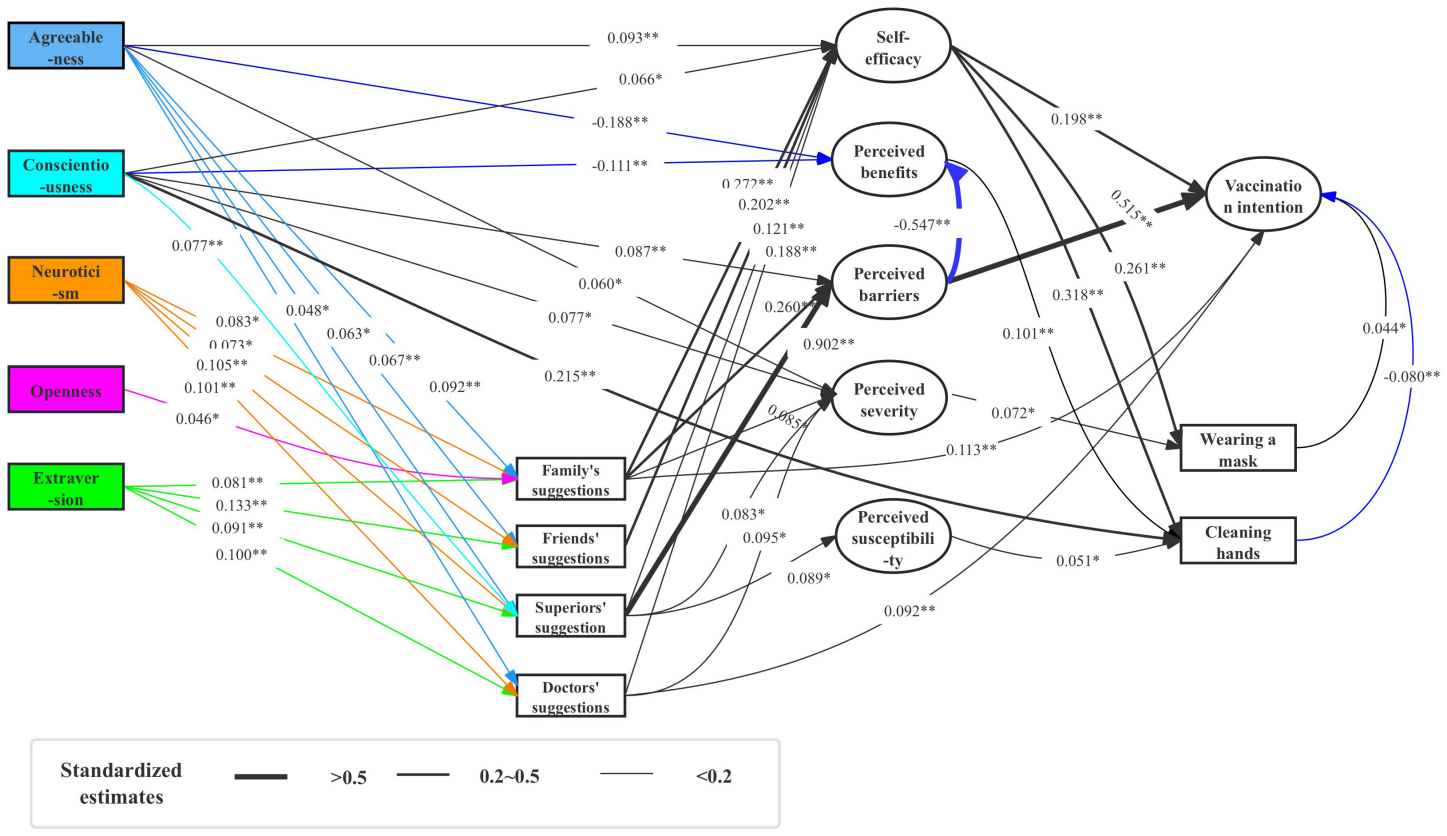
SEM of FFM and HBM variables. ^**^p < 0.001, ^*^p < 0.05.

The structural equation model was established based on Spearman's correlation coefficient matrix (Figure 2). The fitting results of the model were as follows: *X*^2^/df = 2.74, CFI = 0.956, TLI = 0.948, RMSEA = 0.029, and SRMR = 0.031, showing that the structural equation model was well-fitted. The structural equation modeling diagram is shown in Figure 3.

The effects of self-efficacy and perceived barriers on vaccination intention are the most pronounced. Specifically, self-efficacy (β = 0.198) and perceived barriers (β = 0.515) exert direct positive influences on vaccination intention.

Cues to action also play a significant role in influencing vaccination intention. Recommendations from family members (β = 0.113) and doctors (β = 0.092) significantly impact vaccination intention. Furthermore, cues to action indirectly affect vaccination intention by influencing constructs of the HBM. Notably, self-efficacy is positively influenced by several factors derived from cues to action, including family suggestions (β = 0.272) and friends' suggestions (β = 0.202), which have the most substantial effects. In contrast, perceived barriers are predominantly influenced by suggestions from superiors (β = 0.902). Additionally, perceived severity is directly affected by family suggestions (β = 0.085), supervisor suggestions (β = 0.083), and doctor suggestions (β = 0.095). Conversely, perceived susceptibility is directly influenced by supervisor suggestions (β = 0.089).

Personality traits exert an indirect influence on vaccination intention by affecting self-efficacy and perceived barriers. For instance, agreeableness positively impacts self-efficacy (β = 0.093) and perceived severity (β = 0.060), while negatively influencing perceived benefits (β = −0.188). Similarly, conscientiousness positively affects both self-efficacy (β = 0.066) and perceived barriers (β = 0.087), but has a negative impact on perceived benefits (β =-0.111).

Moreover, personality traits directly influence cues to action. This is evidenced by the direct effects of agreeableness (β = 0.092, β = 0.067, β = 0.063, β = 0.048), neuroticism (β = 0.083, β = 0.073, β = 0.105, β = 0.101), and extraversion (β = 0.081, β = 0.133, β = 0.091, β = 0.100) on recommendations from family members, friends, superiors and doctors. Notably, agreeableness has the most significant impact on suggestions from family, while neuroticism exerts the greatest influence on suggestions from superiors and doctors. Conversely, extraversion has the most pronounced effect on suggestions from friends.

Additionally, personality traits directly influence non-pharmaceutical protective behaviors. Specifically, conscientiousness demonstrates a positive direct effect on handwashing behavior (β = 0.215), underscoring its importance in promoting health-related actions.

## 4 Discussion

### 4.1 Effect of HBM constructs on vaccination intention

The exploration of Health Belief Model (HBM) constructs reveals significant associations with public vaccination intentions, aligning with findings from previous studies (Fall et al., 2018; Ernsting et al., 2013). Key factors such as perceived benefits, perceived barriers, self-efficacy, and governmental recommendations have been identified as influential in shaping public acceptance of vaccines (Chen et al., 2021). For instance, a study conducted in Malaysia highlighted that higher perceived benefits, lower perceived barriers to vaccine uptake, and greater perceived susceptibility to infection are critical predictors of individuals' willingness to receive the COVID-19 vaccine. Therefore, interventions designed to target HBM constructs may effectively enhance vaccine uptake (Wong et al., 2020).

Within this study, self-efficacy, perceived barriers, and suggestions from family and healthcare professionals emerged as direct influences on future vaccination intentions. Consistent with prior research on influenza vaccination, self-efficacy was found to significantly impact vaccination intention (Fall et al., 2018; Ernsting et al., 2013). As a crucial determinant of willingness, self-efficacy serves as a noteworthy predictor of regular future vaccination among the general public. Furthermore, it also plays a vital role in promoting non-pharmaceutical protective behaviors, such as frequent mask-wearing and hand hygiene. Given the current climate of vaccine hesitancy, enhancing self-efficacy can significantly encourage non-pharmaceutical protective behaviors, especially when public concerns about vaccine safety, side effects, transportation, or time are present.

Additionally, self-efficacy mediates the effects of cues to action—such as suggestions from family, friends, superiors, and doctors—on vaccination intention. Therefore, public self-efficacy regarding vaccination can be bolstered through supportive suggestions from family and friends, as well as informative guidance from healthcare providers. Family and friends can share their vaccination experiences, while healthcare professionals can provide essential immunization information and reinforce the perceived effectiveness of vaccines (Kempe et al., 2015).

Perceived barriers also exert a significant influence on future vaccination intentions. Concerns about vaccine safety and potential side effects can substantially affect individuals' decisions to vaccinate. The rapid development and rollout of COVID-19 vaccines—taking just over a year from research to deployment (Fisher et al., 2020; Reno et al., 2021)—stand in stark contrast to the typical vaccine development timeline, which usually spans several years. This expedited timeline may contribute to public hesitancy regarding vaccination (Li et al., 2022). However, as the duration of vaccine research and clinical trials increases, the production of vaccines utilizing more established technologies and methodologies may enhance public acceptance.

### 4.2 Effect of personality traits on cues to action

Agreeableness, neuroticism, and extraversion significantly influence cues to action, particularly when suggestions arise from family members, friends, leaders, or doctors. Individuals with higher levels of agreeableness tend to place greater trust in others than in themselves. As depicted in the Structural Equation Model (SEM; Figure 3), agreeableness directly affects the advice received from family, friends, superiors, and doctors, with family members exerting the most substantial influence. This finding suggests that interventions aimed at individuals with high agreeableness may be more effective when they involve familial engagement rather than relying solely on other sources of influence.

Similarly, neuroticism directly impacts the reception of advice from family, friends, superiors, and doctors, with superiors having the most pronounced effect, closely followed by doctors. This could be attributed to the heightened susceptibility of emotionally unstable individuals—often characterized by neuroticism—to adverse external influences (Costa, 1995). Consequently, advice from authoritative figures, such as leaders or healthcare professionals, may resonate more effectively with this demographic.

Extraversion, on the other hand, plays a pivotal role in how individuals respond to suggestions from friends. Extraverts are naturally interactive and adept at absorbing new information through communication and social exchange, making them particularly receptive to advice from their peers. As a result, those with pronounced extraversion profiles are also more open to suggestions from family members.

Lastly, individuals with higher conscientiousness scores are more inclined to adopt non-pharmacological protective behaviors, driven by their inherent caution. This trait instills greater confidence in existing, effective non-pharmacological measures, potentially leading them to adopt a more cautious approach to vaccination.

This study elucidates the significant role of personality traits in shaping vaccination intentions, revealing that preferences for recommendations vary based on traits: agreeableness favors family advice, conscientiousness leans toward supervisors, neuroticism prefers healthcare providers, openness is drawn to family input, and extraversion seeks friends' suggestions. These findings diverge from previous research and uniquely integrate personality traits with cues to action, demonstrating their indirect influence on vaccination intentions. Additionally, the study offers a novel perspective for future vaccination interventions by suggesting that programs consider individual personality profiles or personality portraits of people in the community and develop tailored health education initiatives aligned with preferences for information sources.

## 5 Limitations

Although this study offers significant insights into the relationship between personality traits and vaccination intentions, there are several limitations. First, the reliance on self-reported data may introduce social desirability bias and recall bias. Additionally, some subscales used in this research, such as the TIPI-C, demonstrated low internal consistency. Therefore, future studies should employ a variety of data collection methods to enhance external validity, as well as further validate and optimize these scales by utilizing more established personality trait measures to improve the reliability of the assessments.

## 6 Conclusion

In conclusion, the validity of the health belief model in exploring the factors influencing vaccination intentions was again validated. Self-efficacy is a critical factor in promoting vaccination-related protective behaviors. This study highlights the role of perceived barriers, which can contribute to public hesitancy about “current” vaccination and positively influence future vaccination intentions. Additionally, cues to action significantly shape behavioral intentions and directly impact critical constructs of the Health Belief Model, including perceived susceptibility, severity, barriers, and self-efficacy. The influence of personality traits on cues to action is noteworthy, with neuroticism linked to authority figure influence, extraversion to peer influence, and agreeableness to familial influence. These findings underscore the necessity of incorporating individual differences into public health policy and vaccination promotion strategies, while future research should explore the effects of diverse personality traits and community-specific profiles on vaccination behaviors to enhance intervention effectiveness.

## Data Availability

The original contributions presented in the study are included in the article/supplementary material, further inquiries can be directed to the corresponding author.

## Appendix

**Table A1:** Measures of health belief model.

**Measures**	**Items**	**Response Scale**
Perceived severity	Item 1: Infection with COVID-19 would cause serious health problems	1 (strongly disagree) to 5 (strongly agree)
	Item 2: Infection with COVID-19 would have a detrimental effect on mental health, leading to anxiety, fear, depression, and other negative emotions	
	Item 3: Infection with COVID-19 would have a severe impact on daily life	
	Item 4: Infection with COVID-19 would affect one's or a family's financial income	
Perceived vulnerability	Item 1: Possibility of infection with COVID-19 when studying and working in the same space as an infected person	1 (not at all) to 5 (certain)
	Item 2: Possibility of infection with COVID-19 if you use the same indoor air purification system as an infected person	
	Item 3: Possibility of infection with COVID-19 if you travel in the same vehicle with an infected person	
	Item 4: Possibility of infection with COVID-19 if you live in the same block of flats with an infected person	
Self-efficacy	Item 1: I will be vaccinated even if I test negative for COVID-19	1 (strongly disagree) to 5 (strongly agree)
	Item 2: I will be vaccinated even if there are no new confirmed cases in my city	
	Item 3: I will get vaccinated even if people around me think it is unnecessary	
	Item 4: I will get vaccinated even if the vaccination facility is far from me	
	Item 5: I will get vaccinated even if I am busy with school or work	
Perceived benefits	Item 1: Vaccination is a very effective way to protect me against COVID-19.	1 (strongly disagree) to 5 (strongly agree)
	Item 2: Vaccination greatly reduces the risk of infection to my family and others around me	
	Item 3: Vaccination helps me to concentrate more on my studies, work, and life	
	Item 4: Vaccination helps to end the outbreak as soon as possible	
Perceived barriers	Item 1: Safety and possible side effects of vaccine	1 (strongly disagree) to 5 (strongly agree)
	Item 2: Vaccination can be psychologically taxing	
	Item 3: Vaccine is also a virus will increase the risk of infection	
	Item 4: Vaccination will take time and effort	
	Item 5: It takes time and effort to acquire and learn about the COVID-19 vaccine	
Vaccine intention	Item 1: If a booster of the COVID-19 vaccine is required in the future, will you get vaccinated?	1 (never) to 5 (certain)
	Item 2: Would you get the “new” vaccine in the future if the virus mutates and government policy recommends it?	
	Item 3: If a mutation of COVID-19 required a “new” vaccine, which was proven safe and effective, and vaccination services were available, would you get it?	
Cues to action	**Item 1**: People vital to me (parents, children, partners, etc.) think I should get the COVID-19 vaccine.	1 (strongly disagree) to 5 (strongly agree)
	**Item 2**: People around me (friends, colleagues, classmates, etc.) think I should get the COVID-19 vaccine.	
	**Item 3**: My superiors at work suggested that I should get vaccinated.	
	**Item 4**: Doctor recommends that I should receive the COVID-19 vaccine.	
Wearing a mask	Item 1: Wear a mask when you go outside.	1 (seldom) to 5, (every day)
	Item 2: Wear a mask when you are in confined spaces or public places.	
Cleaning hands	Item 1: Wash your hands before eating any food.	
	Item 2: Wash and sterilize your hands when you get home from outside immediately.	
	Item 3: Sterilize and wipe everything you take home from outside.	
